# Unexpected online gambling disorder in late-life: a case report

**DOI:** 10.3389/fpsyg.2015.00655

**Published:** 2015-05-27

**Authors:** Anne Sauvaget, Susana Jiménez-Murcia, Fernando Fernández-Aranda, Ana B. Fagundo, Laura Moragas, Ines Wolz, Misericordia Veciana De Las Heras, Roser Granero, Amparo del Pino-Gutiérrez, Marta Baño, Eva Real, Maria N. Aymamí, Marie Grall-Bronnec, José M. Menchón

**Affiliations:** ^1^Addictology and Liaison Psychiatry Department, Nantes University HospitalNantes, France; ^2^Department of Psychiatry, University Hospital of Bellvitge-IDIBELLBarcelona, Spain; ^3^CIBER Fisiopatología Obesidad y Nutrición, Instituto de Salud Carlos IIIBarcelona, Spain; ^4^Department of Clinical Sciences, School of Medicine, University of BarcelonaBarcelona, Spain; ^5^Department of Neurology, University Hospital of Bellvitge-IDIBELLBarcelona, Spain; ^6^Departament de Psicobiologia i Metodologia de les Ciències de la Salut, Universitat Autònoma de BarcelonaBarcelona, Spain; ^7^Department of Public Health, Mental Health and Perinatal Nursing, University School of Nursing, University of BarcelonaBarcelona, Spain; ^8^CIBER Salud Mental, Instituto de Salud Carlos IIIBarcelona, Spain

**Keywords:** gambling disorder, online gambling, elderly, late-life, medical condition, behavioral addictions

## Abstract

**Background:** The lifetime prevalence of problem or Gambling disorder (GD) in the elderly (i.e., those over 60 years old) is reported to range from 0.01 to 10.9%. Research has identified several specific risk factors and vulnerabilities in the elderly. Since the late 1990s, an increase in online GD has been observed in the youth population, whereas casinos, slot machines, and bingo seem to be the activities of choice among the elderly. Interestingly, online GD has not been described in the elderly to date.

**Case Description:** We report an 83-year-old man who started online casino gambling from the age of 80 years, leading to debts that exceeded €30,000. He underwent a full clinical and neuropsychological assessment, without any evidence of cognitive impairment or any associated neurodegenerative disease. However, he had risk factors for GD, including adjustment disorder, stressful life events, previous offline casino GD when 50 years old, and dysfunctional personality traits. The change to online GD may have been due to his isolation, movement difficulties, and his high level of education, which facilitated his access to the Internet. Care management focused on individual cognitive-behavioral therapy.

**Conclusion:** The prevalence of online GD may be underestimated among the elderly, and may increase among isolated old people with movement difficulties and ready access to the Internet. However, late-life GD should be considered a diagnosis of elimination, requiring a full medical, psychiatric (including suicide risk), and cognitive assessment. Specific therapeutic approaches need to be proposed and developed.

## Introduction

### Prevalence of gambling disorder in the elderly

Gambling disorder (GD) is the persistence and recurrence of problematic gambling behavior, leading to clinically significant impairment or distress. The lifetime prevalence of GD is estimated at around 0.4–1.0% (American Psychiatric Association, 2013), while that in the elderly (over 60 years old) ranges from 0.01 to 10.9% (Subramaniam et al., 2015), depending on the region, the scale of survey, and the population studied (Hirsch, 2000; McNeilly and Burke, 2000; Bazargan et al., 2001; Wiebe and Cox, 2005). GD among older adults is lower than that among young adults, but it remains an important problem (Grant Stitt et al., 2003; Desai et al., 2004; Vander Bilt et al., 2004; Wiebe and Cox, 2005). In the elderly, the prevalence of GD seems to rise with increasing age, being approximately 1.2% for people over 55 years old (Philippe and Vallerand, 2007) and 3.8% for those over 60 years old (Erickson et al., 2005). Similarly, the prevalence of at-risk gambling seems to increase with age, being 6.4% for those over 60 years old (Erickson et al., 2005). Although most of the studies were performed in a western context (Subramaniam et al., 2015), few studies have explored the prevalence of GD in non-western populations (Tse et al., 2013) and the differences of GD between native and the immigrat population (Lai, 2006; Penelo et al., 2012; Patterson-Silver Wolf Adelv Unegv Waya et al., 2014). For example, an epidemiological study on the prevalence of GD in Singapore revealed that more than 69% of people over 55 years old had gambled in the last 12 months, and that 2.2% of them had evidence of problem gambling (Tse et al., 2013). Furthermore, individuals with problem gambling are likely to start gambling at a young age (Tse et al., 2013).

### Risk factors of GD in the elderly

Overall, older adults share several risk factors with younger people (Ladd et al., 2003; Southwell et al., 2008). Common risks include male sex (Pietrzak et al., 2007), being single, divorced, or separated (Pietrzak et al., 2007), having a low income (Zaranek and Lichtenberg, 2008; Martin et al., 2011), emotional vulnerabilities (Blaszczynski and Nower, 2002), stressful life events (Lee et al., 2012), and having physical or psychological health problems (Pilver et al., 2013). However, other generic risk factors and correlates commonly in the general adult population may not necessarily apply to late-life problem gamblers (McNeilly and Burke, 2002; Grant et al., 2009). Indeed, there are some specific risk factors for GD in the elderly, which underlie a specific vulnerability (Blaszczynski and Nower, 2002; Granero et al., 2014). The risk factors for GD among old adults may be understood from an ecological perspective, in which environmental variables interact with individual characteristics.

From an environmental perspective, risk factors include a lack of support from family and social networks (Zaranek and Lichtenberg, 2008), social gambling environments, poor social adjustment (Pietrzak et al., 2007), stressful life experiences (widowhood and retirement) (Bazargan et al., 2001), frequency and intensity of gambling behavior, and large losses or gains in the first gambling experiences (Weatherly et al., 2004). Petry (2002) found that, contrary to lifetime problem gambling, late-life problem gambling was more associated with employment problems than social, legal, and substance use disorders (Petry, 2002). Race and ethnicity have also been identified as risk factors for GD (Raylu and Oei, 2004; Johansson et al., 2009; Subramaniam et al., 2015). While some studies indicate that certain cultural groups may be more vulnerable to have GD in the elderly, such as African-Americans (Alegria et al., 2009) and Native Americans (Patterson-Silver Wolf Adelv Unegv Waya et al., 2014) in USA, or Chinese in Canada (Lai, 2006), others found that both immigrant and native-born cohorts shared more similarities than differences in their gambling profiles (Penelo et al., 2012).

From a psychological perspective, several personality traits have been implicated. For example, personalities that are characterized by elevated levels of impulsivity and sensation seeking; deficits in coping strategies and problem solving; emotional disturbances such as worry, anxiety, tension, anger, feelings of being slighted; victimization; vulnerability to stress or low self-esteem; and lack of optimism (Zaranek and Lichtenberg, 2008). Older adults may also gamble more in an effort to ameliorate negative emotional states (Subramaniam et al., 2015). Being an old woman may confer a similar or even higher risk of GD than being an older man (Petry, 2002; Blanco et al., 2006).

Studies suggest that GD is highly associated with greater physical and mental health comorbidities (Erickson et al., 2005; Pietrzak et al., 2007; Zaranek and Lichtenberg, 2008; Lorains et al., 2011; Chou and Cheung, 2013). These include major depression (Pietrzak et al., 2007), anxiety disorders (Grant et al., 2009), personality disorders (Pietrzak et al., 2007), and even other addictive disorders, such as alcohol (Desai et al., 2007) and drug abuse (Kessler et al., 2008). These findings are moderated by other studies reporting that elderly subjects with GD are less likely to report anxiety due to gambling and daily tobacco use, and are less likely to have a lifetime drug problem (Potenza et al., 2006).

From a neurobiological perspective, cortical modifications in the elderly, especially in the frontal areas, may have a significant impact on gambling behavior (McCarrey et al., 2012). Previous studies have demonstrated that pathological gamblers show a dysfunctional executive profile characterized by deficits in cognitive flexibility, inhibition response, planning, and decision-making (Goudriaan et al., 2006; Lawrence et al., 2009; Brevers et al., 2014). Moreover, genetic predispositions may explain the higher risk of cognitive flexibility difficulties in pathological gamblers (Fagundo et al., 2014).

### Increased prevalence of online gambling

Since the late 1990s, an increase in online gambling (OG) has been observed, mainly in the young population (Jiménez-Murcia et al., 2011, 2014; Bonnaire, 2012; Granero et al., 2014). Importantly, some studies show substantially higher rates of problem gambling among online gamblers compared with traditional gambling, with rates between 1 and 13% (Wood and Williams, 2011). The favored gambling activities of the elderly seem to be casinos, slot machines, and bingo (McCarrey et al., 2012; Tse et al., 2012). Gambling is also growing as a social activity among the elderly (McNeilly and Burke, 2002; Zaranek and Chapleski, 2005), with motivations for gambling driven by the need for entertainment and leisure than for the money and rewards (Martin et al., 2011). Nevertheless, since older adults are less familiar with new technologies than younger adults, OG in the elderly may appear in the future.

Interestingly, OG in the elderly has not yet been described in the literature. Furthermore, in our daily clinical practice, elderly patients over the age of 80 years are rare. Therefore, we report the first case of an 83-year-old man who developed an OG in later-life. After describing this case in detail, we discuss the possible associated comorbidities, risk factors, and psychopathological explanations before proposing some therapeutic implications.

## Case description

Mr X was an 83-year-old widower living at home near his family. He was retired after a business career. Some family members died very early in his life. A family psychiatric history only uncovered that a distant relative had committed suicide. His medical history included high blood pressure, thrombophlebitis, hypercholesterolemia, and local rectal cancer. The latter had been treated by surgery, chemotherapy, and radiotherapy, 1 year before he presented with GD; although he was still under follow-up surveillance, he was considered to be in remission. He was currently being treated once daily with 50 mg captopril, 50 mg chlorthalidone, and 10 mg simvastatin. He could mobilize with a cane.

His GD history began 30 years prior to this presentation. He reported a 5-year period of gambling in casinos that coincided with economic problems. His OG began when he was 80 years old after he lost his wife, which has led to the accumulation of debts that he has been hiding from his family. His OG problem deteriorated soon after it started, with negative emotional states (feelings of loneliness) and the need to “chase losses.” At the time he presented to us, he had accumulated debts of approximately €30, 000 and he needed several credit cards to cover debts payment. The main gambling problem, for which he consulted, was playing online casinos. His family asked for treatment and he consented to undergo clinical and neuropsychological assessment. On examination, he was not confused and had no obvious symptoms of neurocognitive impairment. He reported feelings of guilt, anxiety, and sleep disorder, but had no suicidal ideation or psychotic symptoms. He denied smoking, drinking coffee or alcohol, and illicit drug use, and we were confident that he was not suffering from any other behavioral addiction.

This case was assessed and treated in the Pathological Gambling Unit (PGU) of the Psychiatry Department of the Bellvitge University Hospital-IDIBELL in Barcelona, Spain. All the results of the assessment are summarized in Tables 1, 2.

**Table 1:** **Diagnosis of gambling disorder according to DSM 5 criteria**.

**DSM-5 MODEL (BASED ON THE GAMBLING DISORDER DIAGNOSTIC CRITERIA)**		
**(A) Persistent and recurrent gambling behavior leading to clinically significant impairment or distress, as indicated by the individual exhibiting four (or more) of the following in a 12-month period**		
1.	Needs to gamble with increasing amounts of money in order to achieve the desired excitement	NO
2.	Is restless or irritable when attempting to cut down or stop gambling	YES
3.	Has made repeated unsuccessful efforts to control, cut back, or stop gambling	YES
4.	Is often preoccupied with gambling	YES
5.	Often gambles when feeling distressed	YES
6.	After losing money gambling, often returns another day to get even	YES
7.	Lies to conceal the extent of involving with gambling	YES
8.	Has jeopardized or lost a significant relationship, job, or educational or career opportunity because of gambling	YES
9.	Relies on others to provide money to relieve desperate financial situations caused by gambling	YES
**(B) The gambling behavior is not better explained by a manic episode**		
Mr X fulfills 8 criteria, with moderate severity		

Table 2**Clinical and neuropsychological assessment results of the case-report**.

**Table:** 

**ClINICAL ASSESSMENT**								
**Test**	**MINI 5.00**	**DQPG-DSM-IV**	**SOGS**	**AUDIT**	**SCL-90-R**	**TCI-R**	**UPPS-P**	**BIS-11**
Results	Adjustment disorder	7	8	0	Somatization = 0.33 Obsessive compulsive = 0.2 Interpersonal sensitivity = 0.44 Depression = 1.07 Anxiety = 0.6 Hostility = 0.5 Phobic anxiety = 0 Paranoid ideation = 0.16 Psychoticism = 0.8 Global Severity Index = 0.57 Positive Symptom Total = 31 Positive Symptom Distress Index = 1.67	Novelty Seeking = 101 Harm Avoidance = 83 Reward Dependence = 83 Persistence = 102 Self-Directedness = 122 Cooperativeness = 130 Self-Transcendence = 93	Urgency = 28 (Lack of) Premeditation = 14 (Lack of) Perseverance = 17 Sensation Seeking = 18 Positive urgency = 37 Total = 114	Attentional = 9 Motor = 17 Non planning = 16 Total = 42

**Table:** 

**NEUROPSYCHOLOGICAL ASSESSMENT**									
**Test**	**MMSE**	**WAIS-III-vocabulary**	**Auditory verbal learning Test**	**Rey-osterrieth Complex Figure Test**	**Semantic fluency**	**Phonemic fluency**	**WAIS-III digit Span**	**Trail making test**	**SCWT**
**Test**	**MMSE**	**WAIS-III-vocabulary**	**Auditory verbal learning Test**	**Rey-osterrieth Complex Figure Test**	**Semantic fluency**	**Phonemic fluency**	**WAIS-III digit Span**	**Trail making test**	**SCWT**
Results	RS = 29/30	RS = 49; SS = 16	Inmediate Recall: 8/15 Delayed Recall: 4/15 Recognition: 13/15	Copy: RS = 31; PC = 99 Recall: RS = 19; PC = 40	RS = 17; PC = 50	RS = 32; PC = 50	RS = 15; SS = 16	Part A: RS = 43; T = 63 Part B: RS = 144; T = 57	RS = 2,50; T = 52
Results	RS = 29/30	RS = 49; SS = 16	Inmediate Recall: 8/15 Delayed Recall: 4/15 Recognition: 13/15	Copy: RS = 31; PC = 99 Recall: RS = 19; PC = 40	RS = 17; PC = 50	RS = 32; PC = 50	RS = 15; SS = 16	Part A: RS = 43; T = 63 Part B: RS = 144; T = 57	RS = 2,50; T = 52

*AUDIT, Alcohol Use Disorders Identification Test; BIS-11, Barratt Impulsiveness Scale; DQPG-DSM-IV, Diagnostic questionnaire for PG according to DSM-IV criteria; MINI 5.00, Mini International Neuropsychiatric Interview; MMSE, Mini Mental State Examination; PC, Percentile; RS, Raw Score; SCL-90-R, Symptom Checklist-90-Revised; SCWT, Stroop Color and Word Test; SOGS, South Oaks Gambling Screen; SS, Standard Score; T, T scores; TCI-R, Temperament and Character Inventory–Revised; UPPS-P, Impulsive Behavior Scale; WAIS, Wechsler Adult Intelligence Scale*.

We conducted a clinical psychological assessment, which included the following: Mini International Neuropsychiatric Interview (MINI 5.00); the South Oaks Gambling Screen (SOGS); the Diagnostic questionnaire for pathological gambling according to DSM-IV criteria (DSM-IV Diagnostic Questionnaire of Stinchfield), the Temperament and Character Inventory–Revised (TCI-R); the Barratt Impulsivity Scale (BIS-11); the Alcohol Use Disorders Identification Test (AUDIT); the UPPS-P Impulsive Behavior Scale; and the Symptom Checklist-90-Revised (SCL-90-R). (See references in Supplementary data).

The clinical assessment therefore highlighted an adjustment disorder. Moreover, Mr X fulfilled DSM5 diagnostic criteria for GD (American Psychiatric Association, 2013) (see Table 1). The psychological assessment confirmed that the patient had a GD (by means of the SOGS and the DSM-IV Diagnostic Questionnaire of Stinchfield), as was already identified in the first clinical interview. It also revealed symptoms of depression, anxiety, hostility, and isolation (measured by the SCL-90-R) (see Table 2).

Regarding personality traits (by means of the TCI-R), the patient was characterized by low scores on harm avoidance, reward dependence, persistence, self-directedness and cooperativeness. However, he showed extremely high scores on the self-transcendence subscale. These results showed that the patient was carefree, courageous, and generally optimistic, although he had difficulties anticipating or preventing potentially harmful or dangerous situations. Moreover, he was critical, skeptical, pragmatic, and individualistic. He did not need the approval of the others to make decisions, and had evidence of detachment and social coldness. Other personality traits indicate a tendency to irresponsibility, instability of purpose, lack of perseverance, and difficulties in planning and organizing goals. Finally, he appeared to be a very spiritual person, which is generally associated with more resources to cope with adversity, illness, suffering, or even death. These features are highly positive in the elderly, as these situations are most likely to happen at this stage of life.

He showed high levels of impulsivity on the impulsivity scales (BIS-11 and UPPS-P). Specifically in the BIS-11, the motor subscale and total score were higher than the means obtained in the Spanish general population (Oquendo et al., 2001). In the UPPS-P, the patient scored high on urgency (negative and positive) and low on (lack of) perseverance, premeditation, and sensation seeking. These results indicated a tendency to act impulsively, both with negative (depression, anxiety or hostility) and positive effects, with a lack of perseverance to achieve goals and meet obligations, and difficulties anticipating the consequences of his behavior. Finally, there was no preference for seeking stimulation or excitement. His AUDIT score was not suggestive of alcohol problems.

To exclude neurocognitive issues, we also performed the following assessments: Mini Mental State Examination-MMSE; Wechsler Adult Intelligence Scale-WAIS-III, subtest of Vocabulary (IQ estimation); Auditory Verbal Learning Test (verbal memory); Rey-Osterrieth Complex Figure Test (visual memory); Animals (semantic fluency); FAS (Phonemic fluency); WAIS-III Digits Span (working memory); Trail Making Test (attention and cognitive flexibility) or TMT; and Stroop Color and Word Test-SCWT (inhibition response). (See references in Supplementary data). The neuropsychological assessment focused on executive functions, memory and verbal fluency. The most important reason for this neuropsychological assessment is that pathological gamblers show a dysfunctional executive profile (Fagundo et al., 2014). Additionally, memory is consistently affected in neurodegenerative diseases and frequently associated with cognitive impairment (Panza et al., 2015). Furthermore, alterations in both qualitative and quantitative aspects of phonemic and semantic fluency have been described in dementias (Fagundo et al., 2008). Thus, considering the age of the patient, the assessment of these cognitive functions was particularly relevant. No cognitive deficits or alterations were observed (see Table 2). To exclude epilepsy, the patient underwent a clinical electroencephalogram, which was normal.

Based on the diagnosis of GD, ambulatory care was implemented based on individual cognitive-behavioral therapy (CBT). The individual CBT consisted of 16 weekly outpatient sessions lasting 90 min each and a 2-year follow-up period. The goal of treatment was to implement CBT strategies to achieve full recovery, defined as the full abstinence from all types of gambling. The general topics addressed in the therapy included psycho-education about the disorder (its course, vulnerability factors, diagnostic criteria, bio-psychosocial models of GD, and phases), stimulus control (money management, avoidance of risk situations, self-exclusion, and changing risky routes), response prevention (alternative and compensatory behaviors), cognitive restructuring focused on the illusions of control over gambling and magical thinking, reinforcement and self-reinforcement, skills training, and relapse prevention techniques. The treatment program has already been described (Jiménez-Murcia et al., 2006) and its short and medium-term effectiveness reported elsewhere (Jiménez-Murcia et al., 2007, 2012). Several family meetings were also conducted to collect a detailed family history and to provide information about individual CBT. Mr X was informed about the intention to publish this case history and he provided signed consent.

## Discussion

Several points warrant further discussion: the originality of the case report, the likely contributing and risk factors, and the therapeutic issues in the case.

**Figure 1:**
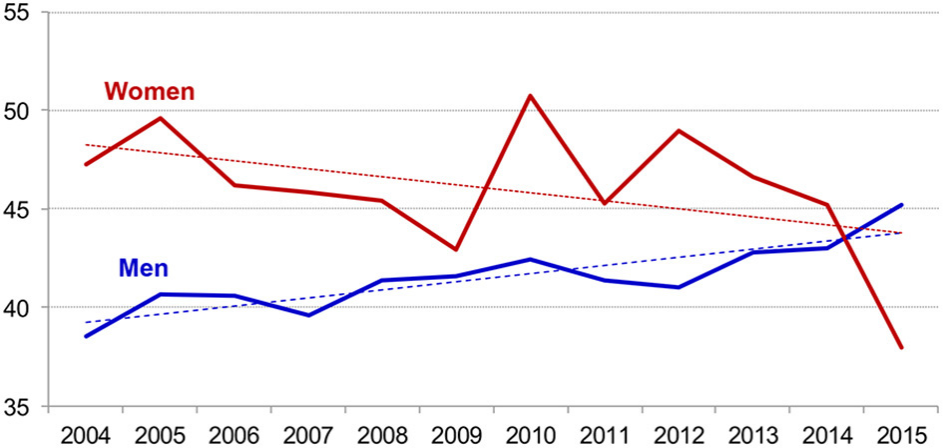
**Evolution of the age means between 2004 and 2015 (*N* = 3.173), in the Pathological Gambling Unit of University Hospital of Bellvitge, Barcelona, Spain**. Dash represents linear trend for each sex.

**Figure 2:**
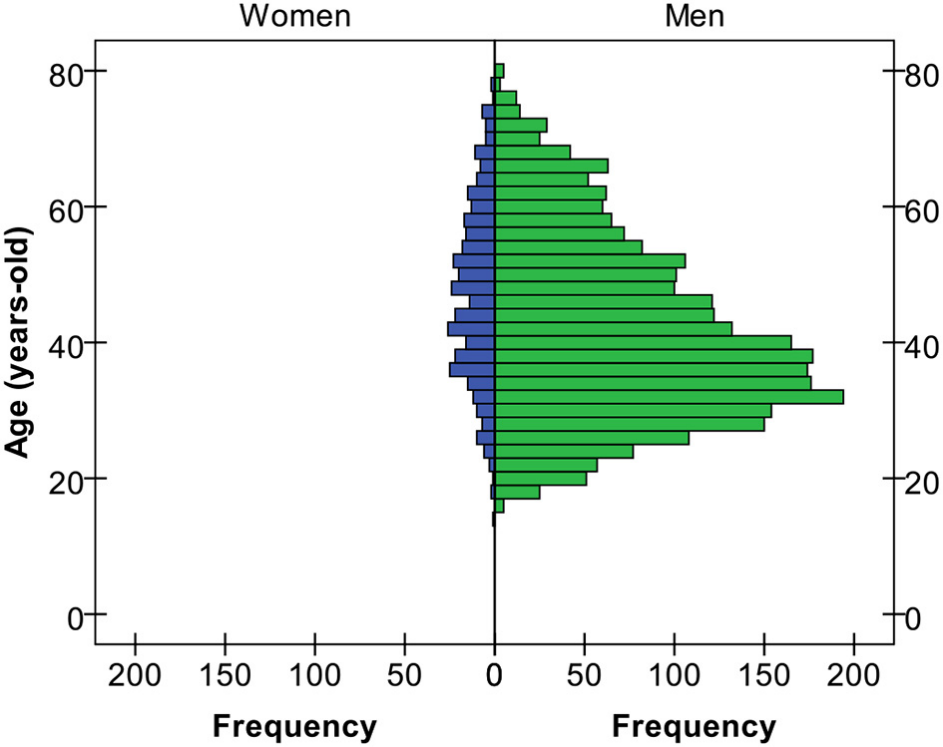
**Age distribution between 2004 and 2015 (*N* = 3.173)**.

First, we believe that a novel feature of our case is the patient's age. Our PG Unit receives over 400 new cases per year. Over the last 10 years, more than 3000 patients have been assessed. We noted a steady increase in the age of the patients, especially in men (see Figure 1). However, the frequency of elderly patients over 65 years old, both men and women, remains low (see Figure 2). Therefore, our case seems particularly rare for several reasons. First his age (83 years) is uncommon. Second, our patient met all the DSM-5 (American Psychiatric Association, 2013) criteria for GD, but the online form was unexpected. OG is commonly found in the young population, probably because young people are more familiar with new technologies. Adjustment difficulties in the elderly may diminish Internet access, while isolation and moving difficulties may facilitate access. Although the elderly are generally assumed to be less aware of new technologies and that they should therefore be less vulnerable to OG, this statement may lose its validity over coming years (see Figure 1). The Internet is already more than two decades old, and the number of users has grown massively in all age groups.

Second, in this case several relevant contributing and risk factors should be taken account and discussed.

Environmental risk factors were evident in this case, with stressful life events both in the near and distant past (the several in his family, and the diagnosis of cancer). He was also socially isolated, even though his family lived very close to him.

Psychological risk factors included the previous history of gambling problems and the evidence of personality traits consistent with GD (Claes et al., 2012; Aymamí et al., 2014; Granero et al., 2014), including high impulsivity and low self-directedness. In fact, this latter dimension is maintained at a low level regardless of age (Granero et al., 2014).

Gambling may have been how this patient coped with anxiety, stress, and negative emotional states. In particular, his OG may have represented a pathological grieving process, allowing him to escape negative feelings. From a constructivist's perspective, research has identified that unresolved losses and mismanagement of stresses are often the most significant predictors of late-life problematic gambling (Tira et al., 2014). This research identified three main pathways that lead to late-life problematic gambling, all linked to a common theme of isolation. In this model, our patient could be considered to have been in the grief pathway, with unresolved losses.

Considering his medical condition, the recent cancer is likely to have strengthened his anxiety and become the second step in the development of GD. This fact is consistent with the literature, suggesting that GD is strongly associated with greater physical and mental health problems (Erickson et al., 2005; Pietrzak et al., 2007; Zaranek and Lichtenberg, 2008; Lorains et al., 2011; Chou and Cheung, 2013; Granero et al., 2014). Interestingly though, except for the entirely understandable adjustment disorder, no other major psychiatric disorders or addictions were evident. This contrasts with the literature, which suggests that major depression and post-traumatic stress disorder are likely explanations for GD (Erickson et al., 2005; Pietrzak et al., 2007; Zaranek and Lichtenberg, 2008; Lorains et al., 2011; Chou and Cheung, 2013; Granero et al., 2014). His history of GD was his greatest risk factor.

The patient had excellent cognitive function for his age. Although this may be a protective factor, high cognitive efficiency may also be a risk factor, facilitating computer literacy and access to the Internet. In the elderly, it is important to keep in mind that a GD could be masking an underlying medical condition, such as frontotemporal dementia (Manes et al., 2010; Kloeters et al., 2013; Ozel-Kizil et al., 2013). Thus, assessing neuropsychological function should be routine in any elderly patients that present with pathological gambling.

Even though our patient had no suicidal ideation or behaviors, suicide risk can result from a GD (Hodgins et al., 2006). This risk is especially pertinent given that older age is a risk factor for completed suicide (Chan et al., 2014). Suicide risk should be systematically and regularly assessed among pathological gamblers, particularly when there is a history of bankruptcy (Wong et al., 2010; Komoto, 2014) or previous suicide attempts (Blaszczynski and Farrell, 1998).

The medications used by our patient are not known to lead to GD. The most likely agent in the elderly population is dopaminergic therapy in Parkinson's disease (Clark and Dagher, 2014; Pirritano et al., 2014). Nevertheless, the medication history should always be checked when assessing a patient with GD, especially in there is an associated psychotic or affective disorder (Gaboriau et al., 2014).

In this case, the patient's history of GD could be considered late-onset given the age of onset and the absence of a psychiatric history (Burge et al., 2004; Desai et al., 2004). However, his current online GD was probably a continuation or relapse of his previous GD. Changing his preference for gambling could have been due to his isolation and movement difficulties, as well as his high educational level, which facilitated access to the Internet. OG, particularly problematic gambling online, was found to be associated with poor mental health and substance use disorder (Scholes-Balog and Hemphill, 2012). OG has several characteristics that potentially make it more attractive and addictive, including its accessibility, anonymity, convenience, feasibility, disinhibition, quickness, simulation, and isolation (Bonnaire, 2012). For these reasons, OG may be a further common form of addiction among old people, probably changing the subtypes of gamblers (Blaszczynski and Nower, 2002; Ledgerwood and Petry, 2006; Álvarez-Moya et al., 2010; Nower et al., 2013; Jiménez-Murcia et al., 2013) and involving prefrontal control (Brand et al., 2014).

Finally, the therapeutic issues must be addressed in a multidisciplinary way with both medical and psychiatric comorbidities being treated. Specific therapeutic approaches and techniques should be proposed for old people, including how to deal with free time, training skills about productive time management, and how to cope with somatic chronic diseases.

## Conclusion

We believe that OG may be an underestimated problem in the elderly due to education levels, shame, and medical and psychiatric disorders. Consequently, new forms of GD may increasingly present to specialist centers. Each patient should undergo medical and psychiatric examination in addition to the specific GD assessment. Indeed, late-life GD should be considered a diagnosis of elimination. Therapeutic approaches that are specific to this population need to be proposed and developed.

### Conflict of interest statement

The authors declare that the research was conducted in the absence of any commercial or financial relationships that could be construed as a potential conflict of interest.
