# Supplementary material for: Unexpected online gambling disorder in late-life: a case report
Source: Front Psychol. 2015 May 27;6:655. doi: 10.3389/fpsyg.2015.00655 (PMC4444736; doi:10.3389/fpsyg.2015.00655)
Supplement: Supplementary file 1 [file DataSheet1.DOCX]

**Supplementary data**

**References of clinical and psychological assessment:**

- Mini International Neuropsychiatric Interview (MINI 5.00) (1), Spanish version (2)
- the South Oaks Gambling Screen (SOGS) (3), Spanish version (4)
- the Diagnostic questionnaire for pathological gambling according to DSM-IV criteria (5), Spanish version (6)
- the Temperament and Character Inventory–Revised (TCI-R) (7), Spanish version (8)
- the Barratt Impulsivity Scale (BIS-11) (9), Spanish version (10)
- the Alcohol Use Disorders Identification Test (AUDIT) (11), Spanish version (12)
- the UPPS-P Impulsive Behavior Scale (13), Spanish version (14)
- the Symptom Checklist-90-Revised (SCL-90-R) (15), Spanish version (16)
- Mini Mental State Examination-MMSE (17)
- Wechsler Adult Intelligence Scale-WAIS-III, subtest of Vocabulary (IQ estimation) (18)
- Auditory Verbal Learning Test (verbal memory) (19)
- Rey-Osterrieth Complex Figure Test (visual memory) (20)
- Animals (semantic fluency) (21)
- FAS (Phonemic fluency) (22)
- WAIS-III Digits Span (working memory) (18)
- Trail Making Test (attention and cognitive flexibility) or TMT (23)
- Stroop Color and Word Test-SCWT (inhibition response) (24)

1. Sheehan D V., Lecrubier Y, Sheehan KH, Janavs J, Weiller E, Keskiner A, et al. The validity of the Mini International Neuropsychiatric Interview (MINI) according to the SCID-P and its reliability. Eur Psychiatry. 1997;12:232–41.

2. Bobes J. A Spanish validation study of the mini international neuropsychiatric interview. Eur Psychiatry. 1998;13:198–9.

3. Lesieur H, Blume S. The South Oaks Gambling Screen (SOGS): A new instrument for the identification of pathological gamblers. Am J Psychiatry. 1987;144:1184–1148.

4. Echeburúa E, Báez C, Fernéndez-Montalvo J, Pérez D. Cuestionario de juego patológico de South Oaks (SOGS): Validación Española. Análisis y Modif Conduct. 1994;20:769–91.

5. Stinchfield R. Reliability, validity, and classification accuracy of a measure of DSM-IV diagnostic criteria for pathological gambling. Am J Psychiatry. 2003;160:180–2.

6. Jiménez-Murcia S, Stinchfield R, Álvarez-Moya E, Jaurrieta N, Bueno B, Granero R, et al. Reliability, validity, and classification accuracy of a spanish translation of a measure of DSM-IV diagnostic criteria for pathological gambling. J Gambl Stud. 2009;25:93–104.

7. Cloninger C. The Temperament and Character Inventory-Revised. Center for. St. Louis (Mo); 1999.

8. Gutiérrez-Zotes JA, Bayón C, Montserrat C, Valero J, Labad A, Cloninger CR, et al. Inventario del Temperamento y el Carácter-Revisado (TCI-R). Baremación y datos normativos en una muestra de población general. Actas Esp Psiquiatr. 2004;32:8–15.

9. Patton JH, Stanford MS, Barratt ES. Factor structure of the Barratt impulsiveness scale. J Clin Psychol. 1995;51:768–74.

10. Oquendo M, Baca-Garcia E, Graver R, Morales M, Montalvan V, Mann J. Spanish adaptation of the Barratt impulsiveness scale (BIS-11). Eur Psychiatry. 2001;15(3):147–55.

11. Babor T, Higgins-Biddle J. C., Saunders J. B. MMG. The Alcohol Use Disorders Identification Test: Guidelines for use in primary care. Geneva World Heal Organ. 2001;1–40.

12. Rubio V, Bermejo J, Caballero M, J S-D. Validation of the Alcohol Use Disorders Identification Test (AUDIT) in primary care. Rev Clin Esp. 1998;198(1):11–4.

13. Whiteside SP, Lynam DR. The five factor model and impulsivity: Using a structural model of personality to understand impulsivity. Pers Individ Dif. 2001;30:669–89.

14. Verdejo-García A, Lozano O, Moya M, Alcázar MA, Pérez-García M. Psychometric properties of a Spanish version of the UPPS-P impulsive behavior scale: reliability, validity and association with trait and cognitive impulsivity. J Pers Assess. 2010;92:70–7.

15. Derogatis L. SCL-90-R. Cuestionario de 90 síntomas. [SCL-90-R. 90-Symptoms Questionnaire]. TEA. Madrid; 1994.

16. González de Rivera JL. Versión española del SCL-90-R [Spanish version of SCL-90-R]. TEA. Madrid, Spain; 2002.

17. Folstein MF, Folstein SE, McHugh PR. “Mini-mental state”. A practical method for grading the cognitive state of patients for the clinician. J Psychiatr Res. 1975;12:189–98.

18. Wechsler D. WAIS-­III administration and scoring manual. Psychol Corp San Antonio, TX. 1997.

19. Rey A. L’examen psychologique dans les cas d'encéphalopathie traumatique. Arch Psychol (Geneve). 1941;28:215–85.

20. Osterrieth PA. Test of copying a complex figure; Contribution to the study of perception and memory. Arch Psychol (Geneve). 1944;30:206–356.

21. Troyer AK, Moscovitch M, Winocur G. Clustering and switching as two components of verbal fluency: evidence from younger and older healthy adults. Neuropsychology. 1997;11:138–46.

22. Henry JD, Crawford JR. A meta-analytic review of verbal fluency performance following focal cortical lesions. Neuropsychology. 2004;18:284–95.

23. Reitan RM. Validity of the Trail Making Test as an indicator of organic brain damage. Percept Mot Ski [Internet]. 1958;8:271–6.

24. Golden C. Stroop Color and Word Test: Manual for Clinical and Experimental Uses. Stoeling. Chicago; 1978.
